# Model-based deep CNN-regularized reconstruction for digital breast tomosynthesis with a task-based CNN image assessment approach

**DOI:** 10.1088/1361-6560/ad0eb4

**Published:** 2023-12-13

**Authors:** Mingjie Gao, Jeffrey A Fessler, Heang-Ping Chan

**Affiliations:** 1 Department of Radiology, University of Michigan, Ann Arbor, MI 48109, United States of America; 2 Department of Electrical Engineering and Computer Science, University of Michigan, Ann Arbor, MI 48109, United States of America

**Keywords:** deep convolutional neural network, digital breast tomosynthesis, image denoising, image reconstruction, microcalcification, model observer, task-based image quality assessment

## Abstract

*Objective*. Digital breast tomosynthesis (DBT) is a quasi-three-dimensional breast imaging modality that improves breast cancer screening and diagnosis because it reduces fibroglandular tissue overlap compared with 2D mammography. However, DBT suffers from noise and blur problems that can lower the detectability of subtle signs of cancers such as microcalcifications (MCs). Our goal is to improve the image quality of DBT in terms of image noise and MC conspicuity. *Approach*. We proposed a model-based deep convolutional neural network (deep CNN or DCNN) regularized reconstruction (MDR) for DBT. It combined a model-based iterative reconstruction (MBIR) method that models the detector blur and correlated noise of the DBT system and the learning-based DCNN denoiser using the regularization-by-denoising framework. To facilitate the task-based image quality assessment, we also proposed two DCNN tools for image evaluation: a noise estimator (CNN-NE) trained to estimate the root-mean-square (RMS) noise of the images, and an MC classifier (CNN-MC) as a DCNN model observer to evaluate the detectability of clustered MCs in human subject DBTs. *Main results*. We demonstrated the efficacies of CNN-NE and CNN-MC on a set of physical phantom DBTs. The MDR method achieved low RMS noise and the highest detection area under the receiver operating characteristic curve (AUC) rankings evaluated by CNN-NE and CNN-MC among the reconstruction methods studied on an independent test set of human subject DBTs. *Significance*. The CNN-NE and CNN-MC may serve as a cost-effective surrogate for human observers to provide task-specific metrics for image quality comparisons. The proposed reconstruction method shows the promise of combining physics-based MBIR and learning-based DCNNs for DBT image reconstruction, which may potentially lead to lower dose and higher sensitivity and specificity for MC detection in breast cancer screening and diagnosis.

## 1. Introduction

Digital breast tomosynthesis (DBT) is a quasi-three-dimensional (3D) breast imaging modality and is becoming a widely used tool for breast cancer detection (Chan *et al* 2014, Gilbert *et al* 2016, Chong *et al* 2019, Gao *et al* 2021b). It improves sensitivity and recall rate in screening as well as diagnostic accuracy because it reduces fibroglandular tissue overlap compared with 2D mammography. However, DBT suffers from noise and blur problems caused by low dose exposure, oblique beam incidence at large projection angles, and source motion in DBT systems with a continuously moving x-ray source (Sundell *et al* 2019, Zheng *et al* 2019). These factors can lower the detectability of subtle signs of cancers such as microcalcifications (MCs) at the early stage. Enhancing the visibility of MCs in DBT without introducing artifacts or noise is a challenge. Our goal is to improve the image quality of DBT in terms of noise and MC conspicuity by model-based reconstruction and deep learning denoising.

DBT reconstruction is an under-determined and ill-posed inverse problem due to the limited-angle scan and incomplete sampling. Iterative reconstruction with or without regularization have been developed for DBT (Zhang *et al* 2006, Sidky *et al* 2009, Das *et al* 2011). Model-based iterative reconstruction (MBIR) is a tomographic reconstruction approach that models the imaging physics and noise statistics and includes a regularization term as a prior of the unknown image (Lange and Fessler 1995, Nuyts *et al* 2013). MBIR has been applied to DBT and has shown good reconstruction quality. For example, Haneda *et al* (2014) used MBIR with regularization to improve the quality of spherical signals in a uniform background. Xu *et al* (2015) employed a Poisson likelihood function and a Gaussian Markov random field prior and improved the detectability of MCs. Zheng *et al* (2018) developed MBIR for DBT by incorporating detector blur and correlated noise (DBCN) modeling and an edge-preserving (EP) regularizer to improve the contrast-to-noise ratio (CNR) and sharpness of MCs.

Medical image denoising and restoration have made remarkable progresses by using deep convolutional neural networks (deep CNNs or DCNNs) (Wolterink *et al* 2017, Yang *et al* 2018, Shan *et al* 2019). This data-driven approach learns the image and noise features from training data for denoising. In the field of DBT, denoising projection views (PVs) before reconstruction was performed using DCNN trained with the mean squared error (MSE) loss (Badal *et al* 2019), the combination of CNR, perceptual, and adversarial loss (Gao *et al* 2020), generative adversarial network (GAN) (Sahu *et al* 2019), and conditional GAN (Gomi *et al* 2022). Gao *et al* (2021a) developed a denoising network for the reconstructed DBT images called DNGAN and improved the MC conspicuity in terms of the detectability index (*d*′) and human observer detection sensitivity (Chan *et al* 2023). Although straightforward and fast, image domain and PV domain denoising methods have limitations because they do not fully exploit measurement statistics and imaging physics.

There is a growing interest in combining DCNN with MBIR (Wang *et al* 2020, 2021). One idea is to unroll the iterative reconstruction loops and replace some steps in the iterations with networks (Yang *et al* 2016, Aggarwal *et al* 2019, Monga *et al* 2021). Teuwen *et al* (2021) applied a learned primal-dual method to DBT reconstruction for breast density and dose estimation. Wu *et al* (2020) unrolled the proximal gradient descent algorithm for DBT reconstruction and achieved better in-depth resolution. Su *et al* (2021) proposed DIR-DBTnet and reduced artifacts. However, all these studies worked on low-resolution or downsampled 3D DBT images because the full-resolution images were too large to fit into memory. Another approach is to use pre-trained denoisers as priors in the reconstruction. Such frameworks include plug-and-play (Venkatakrishnan *et al* 2013) and regularization by denoising (RED) (Romano *et al* 2017). These methods take advantage of the DCNN denoisers for denoising between data-consistency steps and allow one to train the denoisers separately to reduce computation compared to end-to-end training. This approach has shown promising results for MRI (Ahmad *et al* 2020), CT (Ye *et al* 2018, He *et al* 2019), and PET (Xie *et al* 2020). In this study, we adopted the RED framework to combine DNGAN and DBCN modeling and proposed a new model-based DCNN-regularized reconstruction (MDR) method for DBT.

Task-based image quality assessment is an approach that evaluates the quality of medical images based on their specific utility for clinical tasks, such as tumor detection, lesion segmentation, or disease classification (ICRU 1996). It aligns with the ultimate goal of medical imaging to support accurate diagnosis and treatment. Model observers have been developed as a surrogate for human observers to provide task-based image quality assessment in research and development stages where systematic and controlled evaluations are required (Barrett *et al* 1993). Their usage offers several advantages over human observers such as consistency, objectiveness, and computational efficiency. They are also more informative than the generic metrics such as MSE or structural similarity (SSIM) which primarily focus on pixel-level differences between images without considering the specific diagnostic goals.

The clinical task of interest of this work is the detection of MC clusters in DBT. For signal-known-exactly detection tasks, channelized Hotelling observer (CHO) and non-prewhitening observer with eye filter (NPWE) are commonly used and have shown good correlation with human observers (Gang *et al* 2011, Solomon and Samei 2016, Petrov *et al* 2019). However, as a signal-known-statistically (SKS) task, MC clusters have various number of MCs, shapes and spatial distributions. Some studies designed CHO and NPWE for MCs but considered either a single spherical MC (Hu and Zhao 2011, Balta *et al* 2019) or an artificial MC cluster with a fixed layout (Michielsen *et al* 2016, Zeng *et al* 2020). Recently, Zhang *et al* (2021) trained a DCNN to approximate the ideal observer for simulated MC clusters in synthetic mammograms. In the current study, we proposed a DCNN model observer called CNN-MC to evaluate the detectability of MC clusters in human subject DBTs, and a DCNN noise estimator called CNN-NE to evaluate the DBT noise levels. We used CNN-NE and CNN-MC as task-based image quality measures to guide the optimization of DBT reconstruction and to compare several reconstruction and denoising methods for DBT.

## 2. Methods and materials

### 2.1. DBT reconstruction

#### 2.1.1. Background of MBIR and DBCN reconstruction

Assume the post-log PV at the $i$th scan angle is ${y}_{i}\in {{\mathbb{R}}}^{M}$ where $M$ is the number of detector pixels, $i=1,\,\ldots ,{\,N}_{p},$ ${N}_{p}$ is the number of scan angles, and the unknown DBT volume is $x\in {{\mathbb{R}}}^{N}$ where $N$ is the number of voxels. In MBIR, image reconstruction is formulated as an optimization problem with the following cost function:

\begin{eqnarray*}\hat{x}=\mathop{{\mathrm{argmin}}}\limits_{x}L\left(x\right)+\beta \cdot R\left(x\right),\end{eqnarray*}

where $L\left(x\right)$ is the negative log-likelihood or data-fit term that measures the fidelity between the measurement and the reconstructed image, $R\left(x\right)$ is the regularization term, and $\beta $ is a regularization parameter. If the measurement noise is additive Gaussian, ${y}_{i}={A}_{i}x+{\varepsilon }_{i},$ ${\varepsilon }_{i}{\sim }{\mathscr{N}}\left(0,{K}_{i}\right),$ where ${A}_{i}\in {{\mathbb{R}}}^{M\times N}$ is the linear system matrix at the $i$th scan angle, ${K}_{i}\in {{\mathbb{R}}}^{M\times M}$ is the noise covariance matrix, then the data-fit term becomes an inverse-covariance weighted Euclidean norm:

\begin{eqnarray*}L\left(x\right)=\frac{1}{2}\displaystyle \sum _{i=1}^{{N}_{p}}{\unicode{x02016}{y}_{i}-{A}_{i}x\unicode{x02016}}_{{K}_{i}^{-1}}^{2},\end{eqnarray*}

where ${\unicode{x02016}v\unicode{x02016}}_{W}^{2}={v}^{{\prime} }{Wv}$ and $^{\prime} $ denotes matrix transpose. Usually, ${A}_{i}$ does not model the detector blur caused by finite detector pixel size, crosstalk, or the light spread in the scintillator of an indirect detector. Zheng *et al* (2018) introduced a detector blur matrix $B\in {{\mathbb{R}}}^{M\times M}$ in front of ${A}_{i}$ and modeled the resulting noise correlation in the covariance matrix ${K}_{i}:$

\begin{eqnarray*}{L}_{\mathrm{DBCN}}\left(x\right)=\frac{1}{2}\displaystyle \sum _{i=1}^{{N}_{p}}{\unicode{x02016}{y}_{i}-B{A}_{i}x\unicode{x02016}}_{{\left(B{K}_{q,i}{B}^{{\prime} }+{K}_{r}\right)}^{-1}}^{2},\end{eqnarray*}

where ${K}_{q,i}\in {{\mathbb{R}}}^{M\times M}$ is the diagonal quantum noise matrix of the $i$th scan angle, ${K}_{r}\in {{\mathbb{R}}}^{M\times M}$ is the diagonal detector readout noise matrix. In the actual implementation, Zheng *et al* (2018) assumed the quantum noise variances and readout noise variances to be constant across all detector pixels, i.e. ${K}_{q,i}={\sigma }_{q,i}^{2}\cdot {\boldsymbol{I}}$ and ${K}_{r}={\sigma }_{r}^{2}\cdot {\boldsymbol{I}}$ where ${\boldsymbol{I}}$ denotes the identity matrix. Let ${{S}_{i}=\left(B{K}_{q,i}{B}^{{\prime} }+{K}_{r}\right)}^{-1/2}.$ Then ${L}_{\mathrm{DBCN}}\left(x\right)$ has the following equivalent form using the prewhitened PV, ${\widetilde{y}}_{i},$ and the DBCN system matrix ${\widetilde{A}}_{i}:$

\begin{eqnarray*}{L}_{\mathrm{DBCN}}\left(x\right)=\frac{1}{2}\displaystyle \sum _{i=1}^{{N}_{p}}{\unicode{x02016}{\widetilde{y}}_{i}-{\widetilde{A}}_{i}x\unicode{x02016}}_{2}^{2},\,{\mathrm{where}\,\widetilde{y}}_{i}={S}_{i}{y}_{i},{\,\widetilde{A}}_{i}={S}_{i}B{A}_{i}.\end{eqnarray*}

Here, ${S}_{i}$ serves as a prewhitening matrix that boosts high frequency signals but also amplifies noise. Zheng *et al* (2018) introduced an EP regularizer to control the noise level in DBCN reconstruction:

\begin{eqnarray*}{R}_{\mathrm{EP}}\left(x\right)=\frac{1}{1+\gamma }\displaystyle \sum _{j=1}^{N}\left(\eta \left({\left[{C}_{\to }x\right]}_{j}\right)+\eta \left({\left[{C}_{\downarrow }x\right]}_{j}\right)+\gamma \left(\eta \left({\left[{C}_{\nearrow }x\right]}_{j}\right)+\eta \left({\left[{C}_{\searrow }x\right]}_{j}\right)\right)\right),\end{eqnarray*}

where ${C}_{\to },{C}_{\downarrow },{C}_{\nearrow },{C}_{\searrow }{{\mathbb{\in }}{\mathbb{R}}}^{N\times N}$ are the finite differencing operators between neighboring pixels along the horizontal, vertical, and two diagonal directions in the DBT slices, $\gamma $ is an adjustable weight in the diagonal directions, and $\eta \left(t\right)={\delta }^{2}\left(\sqrt{1+{\left(t/\delta \right)}^{2}}-1\right)$ is the hyperbola potential function.

#### 2.1.2. DNGAN denoising

The DNGAN denoiser is a DCNN with trainable weights designed to denoise DBT images (Gao *et al* 2021a). DNGAN was trained with a supervised approach using pairs of noisy DBT image and target low-noise image of the same objects. In the training stage, the denoiser took pairs of image patches extracted from the two sets of DBT slices as input and target output and learned to produce the denoised image patch. The denoiser training loss was a weighted combination of the MSE loss and adversarial loss. The adversarial loss was derived from the Wasserstein GAN with gradient penalty (Gulrajani *et al* 2017) where the denoiser acted as the generator. We implemented the discriminator in the GAN as a trainable VGG-Net (Simonyan and Zisserman 2015). After training, the DNGAN denoiser can be deployed to full DBT slices of any size since it is fully convolutional. The performance of DNGAN denoiser was validated with both phantom and human subject DBT (Gao *et al* 2021a).

#### 2.1.3. Model-based DCNN-regularized reconstruction (MDR)

We integrated the DNGAN denoiser into the iterative reconstruction loop in the general reconstruction optimization problem (1). RED defines a regularizer based on a general image filter or denoiser (Romano *et al* 2017), which in this work is the trained DNGAN denoiser denoted as $G:$

\begin{eqnarray*}{R}_{\mathrm{RED}}\left(x\right)=\frac{1}{2}{x}^{{\prime} }\left(x-G\left(x\right)\right).\end{eqnarray*}

This regularizer promotes the cross-correlation between the residual after denoising and the image, or the residual itself, to be small.

By combining the DBCN data-fit term, the EP regularizer, and the RED regularizer with DNGAN denoiser, we formulated the overall optimization problem for the proposed model-based DCNN-regularized reconstruction (MDR):

\begin{eqnarray*}\hat{x}=\mathop{{\mathrm{argmin}}}\limits_{x}{L}_{\mathrm{DBCN}}\left(x\right)+{\beta }_{\mathrm{EP}}\cdot {R}_{\mathrm{EP}}\left(x\right)+{\beta }_{\mathrm{RED}}\cdot {R}_{\mathrm{RED}}\left(x\right),\end{eqnarray*}

where ${\beta }_{\mathrm{EP}}$ and ${\beta }_{\mathrm{RED}}$ are the regularization parameters.

A variety of optimization algorithms exist for inverse problems with ${R}_{\mathrm{RED}}\left(x\right)$ (Romano *et al* 2017, Reehorst and Schniter 2019). We used the RED proximal gradient method (Reehorst and Schniter 2019) to solve (7). It introduces a secondary image variable $z\in {{\mathbb{R}}}^{N}$ and updates the primary image variable $x$ and the secondary image variable $z$ alternately:

\begin{eqnarray*}{x}_{n}={\mathop{{\mathrm{argmin}}}\limits_{x}{L}_{\mathrm{DBCN}}\left(x\right)+{\beta }_{\mathrm{EP}}\cdot {R}_{\mathrm{EP}}\left(x\right)+\frac{{\beta }_{\mathrm{RED}}}{2}\unicode{x02016}x-{z}_{n-1}\unicode{x02016}}^{2}\end{eqnarray*}

\begin{eqnarray*}{z}_{n}=G\left({x}_{n}\right),\end{eqnarray*}

where $n=1,\,\ldots ,{\,N}_{\mathrm{iter}}$ is the iteration index. Both ${L}_{\mathrm{DBCN}}\left(x\right)$ and ${R}_{\mathrm{EP}}\left(x\right)$ are convex and differentiable in $x.$ We used the diagonally preconditioned gradient descent with ordered subsets for the inner minimization problem (8):

\begin{eqnarray*}{x}_{n}^{i+1}={x}_{n}^{i}-\alpha \cdot P\left({N}_{p}\cdot {\widetilde{A}}_{i}^{\prime} \left({\widetilde{A}}_{i}{x}_{n}^{i}-{\widetilde{y}}_{i}\right)+{\beta }_{\mathrm{EP}}\cdot {\mathrm{\nabla }}{R}_{\mathrm{EP}}\left({x}_{n}^{i}\right)+{\beta }_{\mathrm{RED}}\cdot \left({x}_{n}^{i}-{z}_{n-1}\right)\right)\end{eqnarray*}

where $\alpha $ is the step size, $n$ is incremented after $i$ goes through ${N}_{\mathrm{inner}}$ cycles of $1,\,\ldots ,\,{N}_{p},$ ${x}_{n}^{0}={x}_{n-1}^{{N}_{\mathrm{inner}}{N}_{p}}.$ We used the following preconditioning matrix $P$ whose inverse majorizes the Hessian of the cost function (8):

\begin{eqnarray*}P={\left(\left(\displaystyle \sum _{i=1}^{{N}_{p}}\frac{1}{{\sigma }_{q,i}^{2}+{\sigma }_{r}^{2}}\mathrm{diag}\left\{{A}_{i}^{{\prime} }{A}_{i}{{\bf{1}}}_{N}\right\}\right)+8{\beta }_{\mathrm{EP}}\cdot {\boldsymbol{I}}+{\beta }_{\mathrm{RED}}\cdot {\boldsymbol{I}}\right)}^{-1},\end{eqnarray*}

where ${{\bf{1}}}_{N}$ denotes the vector of ones of length $N.$

### 2.2. Task-based image quality measures

#### 2.2.1. CNN MC classifier (CNN-MC)

Clustered MCs are one of the important signs of early breast cancer and image noise can negatively impact its diagnosis. The detectability of MC clusters and the image noise are therefore important indicators of image quality for DBT reconstruction. We developed an MC classifier as a model observer for the detection task of differentiating breast structured background with and without clustered MCs in human subject DBTs for image quality evaluation. Importantly, reconstruction or other image processing processes can inadvertently enhance artifacts mimicking calcifications in the normal tissue background. Image quality assessment by MC classifiers takes into account the false positives (FPs) while other measures such as the detectability index (*d’*) focus only on the visibility of the target objects. The MC classifier was implemented as a DCNN with trainable weights, and therefore called CNN-MC. It took an image region of interest (ROI) or patch as input. The training set included MC patches as positives with label of 1 and MC-free background patches as negatives with label of 0. The training loss was the binary cross entropy (BCE) loss, which has been shown to give the maximum likelihood estimation of the DCNN weights from the training data (Kupinski *et al* 2001). The CNN-MC output a score between 0 and 1 indicating the likelihood that an input patch contained clustered MCs. After training, the CNN-MC model with frozen weights was applied to the test patches.

We trained the CNN-MC for each image condition and tested it accordingly. The area under the receiver operating characteristic (ROC) curve (AUC) of the test set scores was used as an MC detectability measure. If the underlying image condition enhanced the MCs effectively, the CNN-MC should learn the MC features more accurately during training and have better classification ability between image patches with and without MCs during testing, resulting in a higher AUC metric. Any MC-like artifacts in the background patches would degrade the classification performance of CNN-MC and reduce the AUC. We studied the use of CNN-MC to rank the relative performances of different DBT reconstruction and denoising methods.

There are variations in the CNN-MC observer modeling. DCNN classifiers with different network structures learn image features differently. Even for the same network structure, the randomness in DCNN training such as kernel initialization or data batching can lead to different local minima. These are akin to the interobserver and intraobserver variabilities of human observers. To account for these variations, we investigated the VGG-Net (Simonyan and Zisserman 2015), the ResNet (He *et al* 2016) and the ConvNeXt (Liu *et al* 2022) of similar sizes, as shown in table 1, as the backbone structures of CNN-MC. We also repeated the training multiple times with different random initialization for each structure. The image condition rankings from the individual models and the final combined rankings were analyzed.

**Table 1.** Network structures of CNN-MC and CNN-NE in this study.

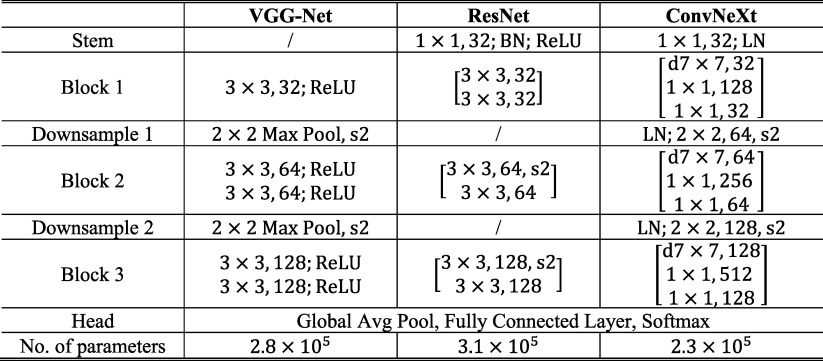

* The input to the models is a 128 × 128 pixel image patch. BN: batch normalization. LN: layer normalization. Convolutional layers are specified by the kernel size and number of filters. ‘d’ denotes the depth-wise convolution. ‘s2’ denotes the stride-2 operation. The brackets denote ResNet block or ConvNeXt block.

#### 2.2.2. CNN noise estimator (CNN-NE)

It is important to assess the image noise for an image processing technique because enhancing the signals is always associated with a change in noise. We developed a DCNN noise estimator (CNN-NE) to quantify the root-mean-square (RMS) noise level of its input image patch. We designed their network structures to be the same as those for CNN-MC (table 1), except for without the exit softmax function, to facilitate transfer learning (discussed next). The training set contained DBT image patches of breast structured background with the training labels calculated by an ROI-based method for each patch as follows: the patch was divided into 10 × 10 pixel ROIs with 25 pixel spacing on a grid, the ROI background trend was removed by a quadratic fitting, the RMS variations of the ROI pixel values were then calculated and averaged over all ROIs as the RMS noise. The CNN-NE was implemented as a regression model. The training loss was the MSE loss between the estimated RMS noise and the training labels. After training, the CNN-NE model was applied to the test set patches. The average of the estimated RMS noise over all patches was used as a noise measure of the entire set.

#### 2.2.3. Transfer learning

For training CNN-NE and CNN-MC, we adopted the training technique of transfer learning (Yosinski *et al* 2014, Samala *et al* 2019). The CNN-NE required only breast structured background images with a range of noise levels so that a large training set could be obtained relatively easily. The trained CNN-NE then served as the pre-trained model to be further fine-tuned as CNN-MC by transfer learning. Since the availability of MC-positive samples was limited, transfer learning reduced the training sample size required and improved the model robustness. It also reused the weights that the model learned from the source task of noise estimation to encourage the CNN-MC to focus on the background noise patterns in the downstream task of MC detection.

### 2.3. Data sets

We modeled three DCNNs: DNGAN, CNN-NE and CNN-MC, for denoising, noise estimation, and MC classification, respectively, in this work. To facilitate the training, validation, and testing of these DCNNs, we prepared two primary DBT sources: virtual phantom DBTs and human subject DBTs. This section first provides an overview of these data sources and how they were used for the data sets, as summarized in table 2, and then delves into the details in the following subsections.

**Table 2.** Summary of data sets for DCNNs.

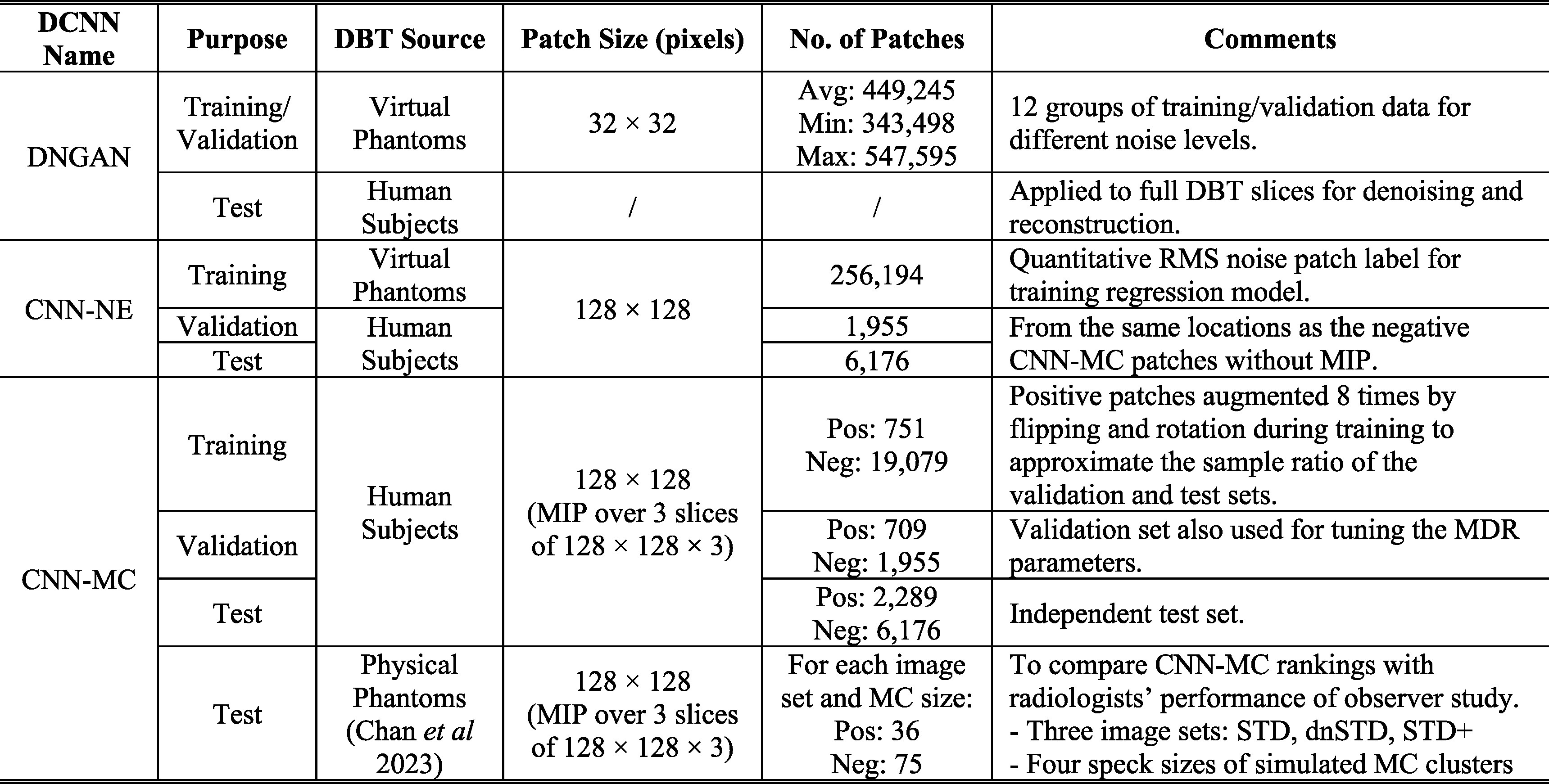

* Pixel size of all patches is 0.1 mm × 0.1 mm.

Gao *et al* (2021a) performed training and validation of DNGAN using virtual phantom DBTs and demonstrated its transferability to human subject DBTs. In the current work, we used the virtual phantom DBTs for the DNGAN training set. This choice was motivated by their flexibility and controllability in generating a wide range of image noise levels with different breast densities and thicknesses. Similar to Gao *et al* (2021a), we chose a small training patch size of 32 × 32 pixels for DNGAN, allowing it to concentrate on local image structures during adversarial training (Isola *et al* 2017). The denoiser was fully convolutional at deployment so that the training patch size would not affect the image sizes for which it could be used.

The training set, validation set and independent test set of CNN-MC were prepared using human subject DBTs because we were interested in evaluating the detectability of MC clusters in real patient data and it is difficult to simulate the wide variations in features of real MC clusters by virtual phantom software. The input patch size of CNN-MC was 128 × 128 pixels. This patch size was chosen to cover typical MC clusters in patient cases while keeping the processing time modest.

The CNN-NE training set was extracted from the virtual phantom DBTs that had a wide noise range, similar to the motivation of the DNGAN training set. For the CNN-NE validation and test sets, we had to use the same human subject DBTs as those for CNN-MC. This enabled us to calculate the RMS noise for each reconstruction, which complemented the AUC metric for MC detectability by CNN-MC. These two CNNs therefore provided the two metrics for the task-based image quality assessment plot, as detailed in the Results section below. The input patch size of CNN-NE was the same as CNN-MC because the two networks shared the same structures for transfer learning.

In addition, Chan *et al* (2023) conducted an observer study with radiologists using a set of physical phantom DBTs with simulated MC clusters to demonstrate the improvement of MC detection by DNGAN denoising. In the current study, we reused their data set to test the capability of CNN-MC in ranking MC detectability relative to human readers. We calculated the relative rankings of the CNN-MC AUC for the MC clusters in the physical phantom DBTs, and then compared them with the relative performance of radiologists’ reading under the image conditions of the observer study.

#### 2.3.1. Virtual phantom DBTs

We generated a collection of virtual breast phantoms at a voxel resolution of 0.05 mm using the anthropomorphic breast model from the Virtual Imaging Clinical Trial for Regulatory Evaluation (VICTRE) package (Badano *et al* 2018). The VICTRE project considered four breast density categories and one compressed thickness for each category. They stated that this design mirrored the cohort demographics of their comparative clinical trial. In our study, we used the same breast density setting as VICTRE, and slightly varied the thicknesses to make it more varied like human data. In particular, a total of 70 phantoms were simulated at a range of glandular volume fractions (GVF), including 10 almost entirely fatty (5% GVF), 10 scattered fibroglandular dense (15% GVF), 25 heterogeneously dense (34% GVF), and 25 extremely dense (60% GVF). The thicknesses of the compressed phantoms for the four density categories were 52–70 mm at every 2 mm, 46–64 mm at every 2 mm, 36–60 mm at every 1 mm, and 31–55 mm at every 1 mm, respectively.

We generated the PVs using the Monte Carlo x-ray imaging simulator (Badal *et al* 2021). We configured the scan geometry as 9 PVs in 25° scan angle and 3.125° increments and the x-ray spectrum as 34 kVp Rh/Ag to model the Pristina DBT system (GE Healthcare, Waukesha, WI). The distances between source and isocenter, isocenter and breast support, breast support and detector were 617 mm, 20 mm, 23 mm, respectively. Scatter and electronic noise were not simulated. The exposure was adjusted for each phantom so that the estimated mean glandular dose matched the measured value for that breast thickness from the automatic exposure control (AEC) of Pristina. To simulate a wide range of noise levels, we repeated the simulations and varied the exposure by factors of 0.25, 0.3, 0.35, 0.4, 0.5, 0.6, 0.75, 1, 1.4, 2, 3. These exposure factors were selected such that the noise standard deviations of the post-log x-ray intensity on the PVs, and hence the reconstructed DBT images, were evenly spaced. Finally, we reconstructed the DBT images using 3 iterations of the simultaneous algebraic reconstruction technique (SART) (Zhang *et al* 2006).

#### 2.3.2. DNGAN training set

For DNGAN training, we used the virtual phantom DBTs with an exposure factor of 3 as the training high dose (HD) targets and the DBTs with the remaining 10 exposure levels as the training low dose (LD) inputs. To ensure that DNGAN covered a wide range of DBT noise levels due to variations in patient sizes and other factors in clinical settings, we trained a suite of DNGAN denoisers, each of which can handle a certain range of image noise levels. To do so, we calculated the average RMS noise for each DBT volume using the method described in section 2.2.2 and obtained the range of the noise values for the entire set of virtual DBT volumes. We empirically grouped the volumes into 12 groups so that the noise interval of each group was reasonably small. We then extracted training patches within the breast regions from each group. This resulted in 12 groups of DNGAN training data with an average of 449 245 (min: 343 498; max: 547 595) LD/HD pairs of 32 × 32 pixel training patches for training a denoiser for each group.

#### 2.3.3. CNN-NE training set

We randomly extracted 128 × 128 pixel patches within the breast regions from all the 70 virtual phantom DBTs and 11 exposure levels to form the CNN-NE training set. This gave a total of 256 194 patches. The low-frequency background was removed from the patches to reduce the nonuniformity caused by heterogeneous breast structures (Chan *et al* 1995).

#### 2.3.4. Human subject DBTs

We had 238 human subject cases with biopsy-proven MCs collected with Institutional Review Board approval and written informed consent. Two-viewed DBTs of the breast with MCs were acquired for each case using the GEN2 prototype DBT system (GE Global Research, Niskayuna, NY). The system acquired 21 PVs in 60° scan angle and 3° increments with a 29 kVp Rh/Rh x-ray beam. The distances between source and isocenter and between isocenter and detector were 640 mm and 20 mm, respectively. The breast support was at the same height as the isocenter. For our reconstructions, we used the central 9 PVs in 24° angle so that the scan geometry was similar to that of the GE commercial Pristina DBT system. The radiation doses at 9 PVs were similar to those of the Pristina system. A Mammography Quality Standards Act (MQSA) approved radiologist marked the biopsied MCs with 3D bounding boxes in the reconstructed DBT images based on all available clinical information.

#### 2.3.5. CNN-MC training set and CNN-NE/CNN-MC validation and test set

**Figure 1.**
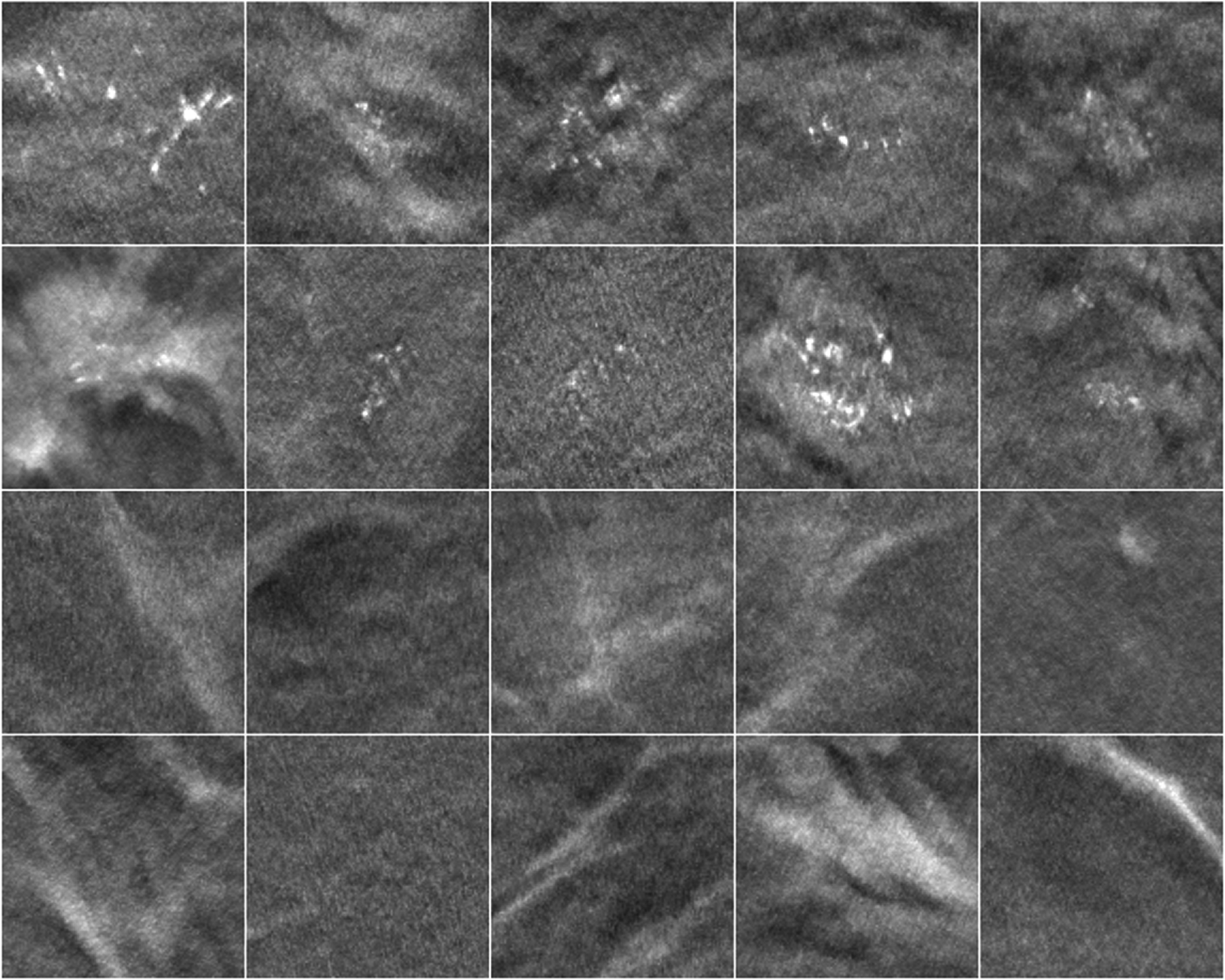
Example 3-slice MIP patches with MCs (top two rows) and without MCs (bottom two rows) from the CNN-MC training set of human subject DBTs. The patch size is 128 × 128 pixels (12.8 mm × 12.8 mm).

We split the human subject DBTs by case into three disjoint sets for training, validation and testing. The training set consisted of 64 cases, or 127 DBT views (one view was lost due to technical issue). The validation set consisted of 52 cases, or 104 DBT views. The remaining 122 cases with 246 views (one case had bilateral MCs) were sequestered as independent test set. For each data set, positive patches of size 128 × 128 × 3 pixels containing clustered MCs were extracted inside the radiologist’s 3D boxes on a regular grid with centers separated by 128 pixels in the two directions along a slice and separated by 1 slice with offset centers along the depth direction. An MC cluster therefore might be extracted multiple times in part or in whole but spatially shifted in each patch, which served as augmented samples to reduce the imbalance between the two classes. Non-overlapping negative patches of the same size were randomly extracted outside the 3D boxes in the structured breast background. For the CNN-MC, we obtained 751 positive and 19 079 negative patches for the training set, 709 positive and 1955 negative patches for the validation set, and 2289 positive and 6176 negative patches for the test set. For each patch, we removed the low-frequency background and took maximum intensity projection (MIP) over the three slices to emphasize the MCs, if any. Figure 1 shows example MIP patches from the CNN-MC training set. For the validation and test sets of CNN-NE, the same sets of negative patches for CNN-MC were extracted except that only the central slices without MIP were used.

#### 2.3.6. Physical phantom DBTs

The physical phantom DBTs were used in a previous observer study with radiologists to detect MC clusters under three image conditions (Chan *et al* 2023). Each of the six breast phantoms were 5 cm thick and made of 50% glandular/50% adipose heterogeneous breast-tissue-equivalent materials. Clusters of simulated MCs of four nominal speck diameters (0.125–0.150 mm, 0.150–0.180 mm, 0.180–0.212 mm, 0.212–0.250 mm) were embedded in the phantoms. There were 36 clusters for each diameter range, giving a total of 144 clusters. The phantoms were imaged twice using two automatic exposure modes of the Pristina DBT system: the standard (STD) mode, which was for routine patient imaging, and the STD+ mode, which used about 54% more dose than that of STD. The DBT images were reconstructed by the built-in commercial algorithm. A third image set called dnSTD was obtained by denoising the STD set using the DNGAN denoiser. The denoiser was trained and validated by Gao *et al* (2021a) using a separate set of physical phantoms that shared the same materials but had different designs from the test phantoms in the Chan *et al* (2023) study. For each image set and each MC speck size, we extracted a total of 36 128 × 128 pixel background-corrected 3-slice MIP patches centered at the MC clusters as positives and paired them with 75 MC-free background MIP patches as negatives for CNN-MC testing. The set of 75 negative patches was extracted from different random locations for each MC positive set.

### 2.4. Implementation

For MDR, the implementation of ${L}_{\mathrm{DBCN}}\left(x\right)$ and ${R}_{\mathrm{EP}}\left(x\right)$ was described in Zheng *et al* (2018). The 12 DNGAN denoisers were trained separately following the process described in Gao *et al* (2021a). We retrained DNGAN using our newly generated training set that had a wider range of noise levels and breast characteristics while keeping all training settings unchanged. When DNGAN was called during reconstruction, the algorithm automatically estimated the RMS noise of the DBT volume at that iteration and selected the denoiser with the closest-matched noise from the 12 trained denoisers for deployment. We chose ${N}_{\mathrm{iter}}$ and ${N}_{\mathrm{inner}}$ to 3 experimentally. Section 3.2 discusses the selections of ${\beta }_{\mathrm{EP}}$ and ${\beta }_{\mathrm{RED}}.$ Both $x$ and $z$ were initialized to 0. The image variable $z$ was saved as the final reconstructed image. All the PVs had a pixel size of 0.1 mm × 0.1 mm. The reconstructed images had a voxel size of 0.1 mm × 0.1 mm × 1 mm and were saved in DICOM format with a pixel value range of $\left[0,4095\right].$ Metal artifact reduction (Lu *et al* 2017) and truncated projection artifact reduction (Lu *et al* 2013) were implemented to minimize these artifacts in the reconstructed DBT volumes.

For the CNN-NE (CNN-MC) training, we set the number of epochs to 600 (300) and the mini-batch size to 2048 (512). The Adam optimizer was set with an initial learning rate of ${10}^{-3}$ (${10}^{-5}$) and dropped by a factor of 0.8 for every 20 (10) epochs. We initialized the kernel weights of CNN-NE randomly and initialized CNN-MC with the trained CNN-NE. In CNN-MC training, the positive patches were augmented by a factor of 8 (4 rotations by 90 degrees and another 4 rotations after flipping). During CNN-MC deployment, we augmented every patch and averaged the 8 output scores as the patch score. We further averaged the patch scores of all positive patches from the same MC cluster by DBT view for the ROC analysis. We repeated the training of CNN-NE and fine-tuning of CNN-MC 5 times. Each repeated experiment used a different random seed for the weight initialization of CNN-NE and the data batching during CNN-NE training and CNN-MC fine-tuning. We reported the mean and standard deviation of the 5 CNN-MC AUCs. We reported only the mean of the 5 CNN-NE RMS estimates because all standard deviations were smaller than 2% of one pixel value.

### 2.5. Image conditions

We compared MDR with other approaches, including SART (Zhang *et al* 2006), SART with multiscale bilateral filtering (MSBF) regularization (Lu *et al* 2015), DBCN reconstruction (Zheng *et al* 2018), RED reconstruction without DBCN modeling, and post-reconstruction DNGAN denoising on SART and DBCN images. Gao *et al* (2023) evaluated DNGAN denoising of PVs for SART reconstruction and observed that it suffered from blurry MCs. Therefore, PV denoising was not considered in this study.

## 3. Results

### 3.1. Effectiveness of DNGAN, CNN-NE and CNN-MC

**Figure 2.**
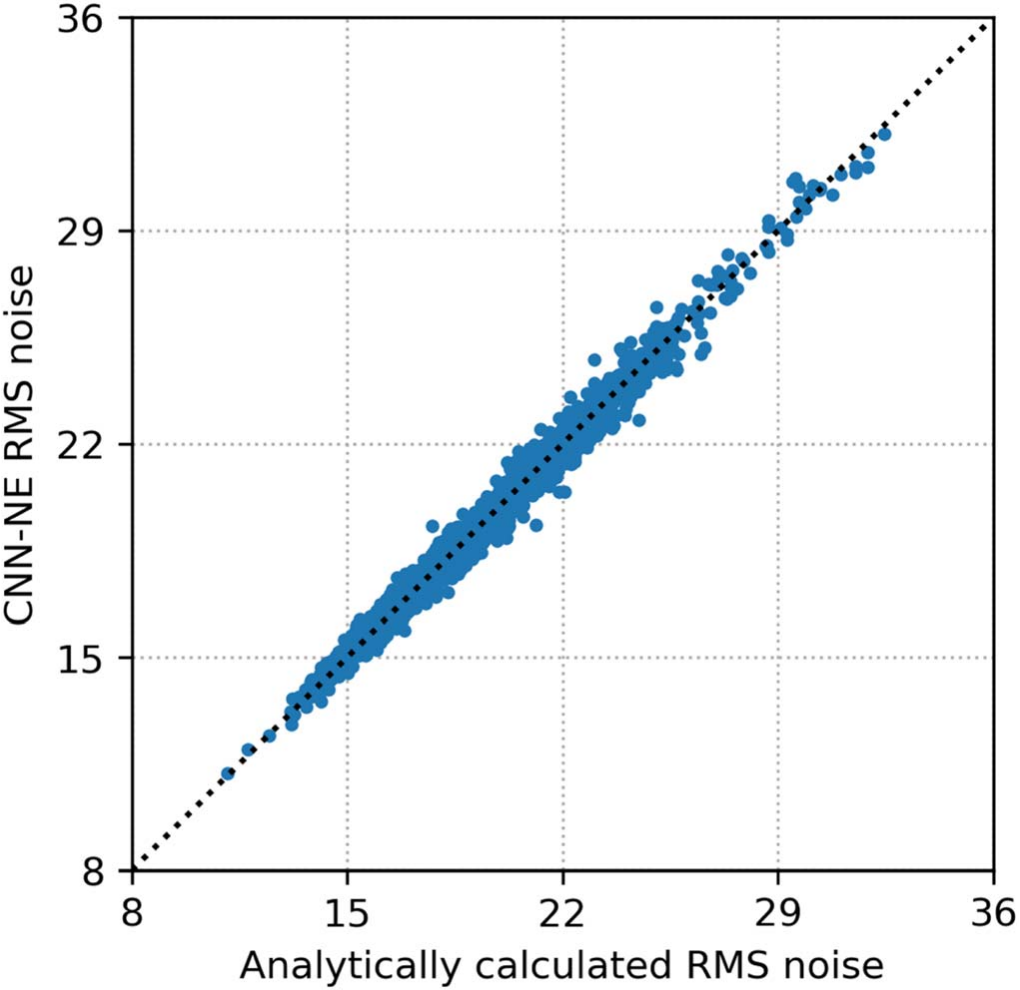
The scatter plot of the CNN-NE estimated RMS noise versus the analytically calculated RMS noise for the individual patches in the CNN-NE validation set (human subject DBT images). The dotted line is the diagonal line. The RMS noise is plotted in terms of pixel values.

As an example, we deployed one of the five CNN-NE models with VGG-Net backbone trained with different random initializations to the CNN-NE validation set from 2 iterations of SART images. Figure 2 shows that the CNN-NE RMS noise of the individual patches correlated well with the analytical calculations (correlation coefficient = 0.993, *p* < 0.0001), indicating that CNN-NE can accurately estimate the RMS noise of an input patch. Similar correlations were observed for other reconstructions (not shown). While CNN-NE was trained using VICTRE images from 3 iterations of SART, it was applicable to other reconstructions and human subject images at deployment because it was designed to assess the RMS variations in noisy pixels without relying on the specific image conditions.

**Figure 3.**
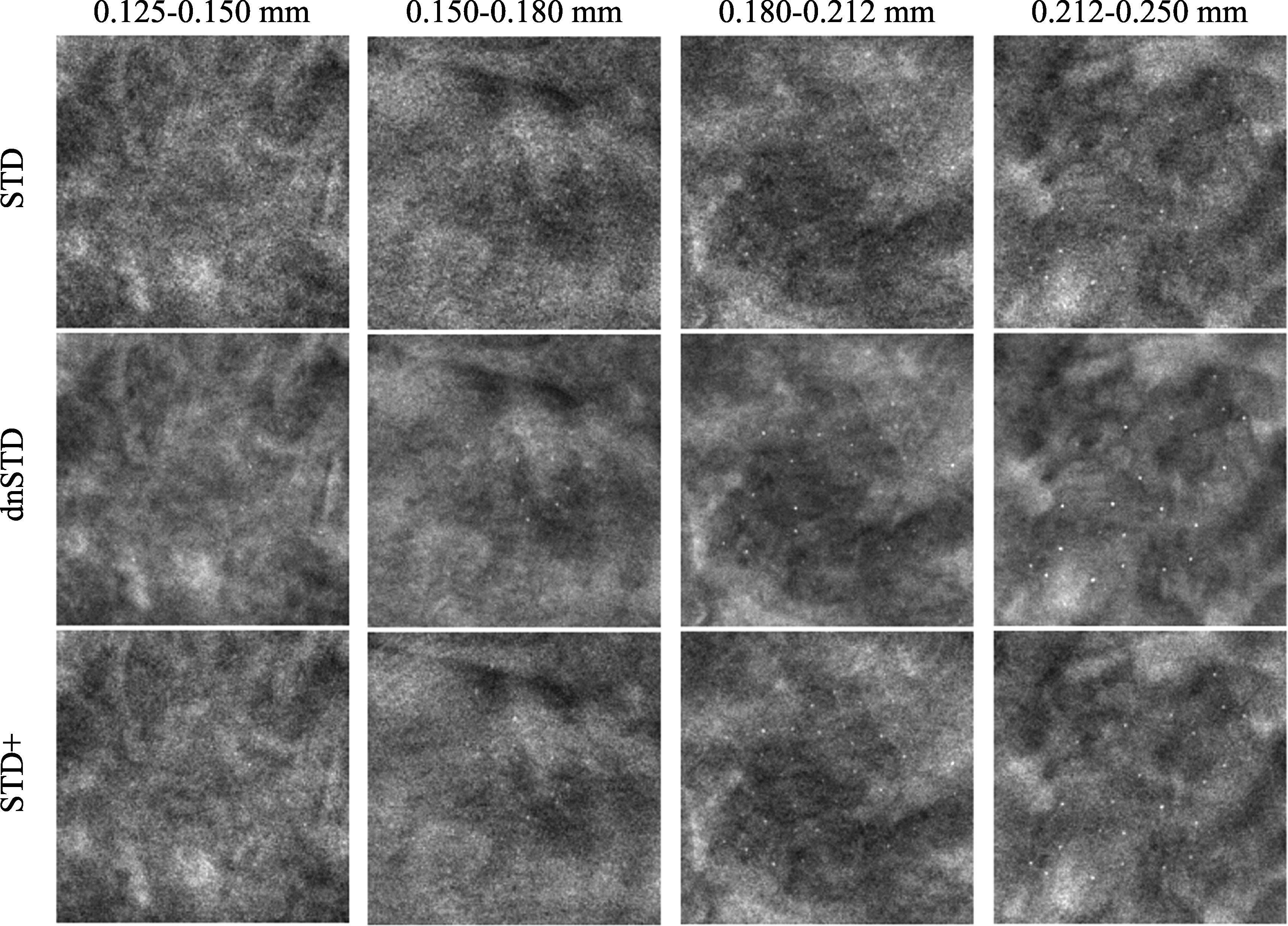
Example images (18 mm × 18 mm) with MC clusters from the physical phantom DBTs acquired and reconstructed by a GE Pristina DBT system. The dnSTD images were obtained by DNGAN denoising on the STD images. The STD+ mode used 54% more dose than the STD mode.

Chan *et al* (2023) conducted an observer study using physical phantom DBTs and had seven radiologists detect the MC clusters in the STD, dnSTD, and STD+ conditions. Figure 3 shows example of the MC clusters. The study found that the average sensitivities of detecting MC clusters in dnSTD, obtained from DNGAN denoising of the STD images, were higher than those in STD for all 4 MC speck sizes and were comparable to those in STD+. This shows the effectiveness of the DNGAN denoiser to reduce noise and enhance MCs in DBT.

**Figure 4.**
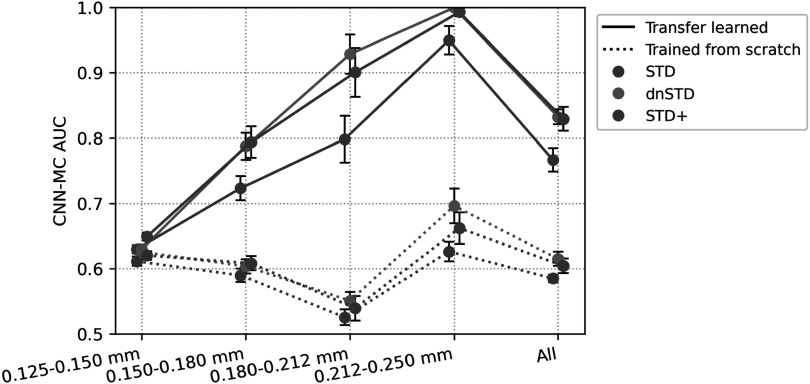
AUCs of CNN-MC for the classification of image patches with and without MC clusters in the physical phantom DBTs of 3 image conditions. The data points and error bars were obtained from the mean and standard deviation of the 5 repeated models trained with different random initialization. The points are slightly shifted horizontally to avoid overlap.

To demonstrate the potential of using CNN-MC in estimating the relative detectability of MC clusters, we applied the CNN-MC models to the extracted patches from the phantom DBTs used in the observer study. The CNN-MC models used VGG-Net backbone and were trained for 2 iterations of SART images. The solid lines in figure 4 show that the AUCs increased with MC speck size. The AUCs of dnSTD were higher than STD and close to STD+. The relative rankings for the speck sizes and the image conditions given by the CNN-MC were consistent with the findings of the observer study. To show the benefit of transfer learning, we repeated the experiment but trained the CNN-MC models from scratch instead of from the pre-trained CNN-NE. In this case, the CNN-MC failed to learn and likely estimated the patch scores based on image features unrelated to MC detectability. This led to very low AUCs and a lack of correlation between MC size and AUC, as shown by the dotted lines in figure 4.

### 3.2. Parameter selection for MDR regularization

**Figure 5.**
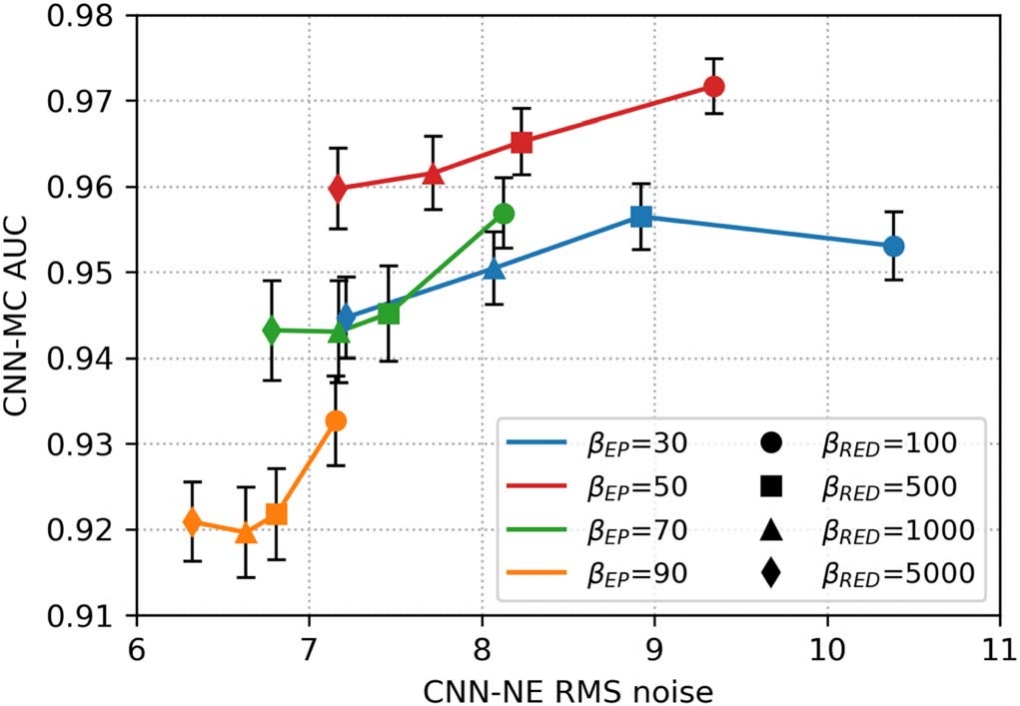
The task-based image quality assessment plot showing the tradeoff between MC cluster detectability (CNN-MC AUC) versus image noise (CNN-NE RMS noise) on the validation set reconstructed with different regularization parameters ${\beta }_{\mathrm{EP}}$ and ${\beta }_{\mathrm{RED}}$ in MDR. The data points obtained with a fixed ${\beta }_{\mathrm{EP}}$ are linked by a solid line and data points for a fixed ${\beta }_{\mathrm{RED}}$ are shown with the same symbol.

To select the regularization parameters ${\beta }_{\mathrm{EP}}$ and ${\beta }_{\mathrm{RED}}$ in MDR, we performed a grid search with ${\beta }_{\mathrm{EP}}$ = 30, 50, 70, 90 and ${\beta }_{\mathrm{RED}}$ = 100, 500, 1000, 5000. Figure 5 shows the task-based image quality assessment plot of CNN-MC AUC versus CNN-NE RMS noise for the VGG-Net backbone on the validation set for the different parameters. At a low ${\beta }_{\mathrm{EP}}$ value of 30, the AUCs were low because the noise was poorly suppressed and thus obscured MC conspicuity. As ${\beta }_{\mathrm{EP}}$ increased to 50, the MC signals were enhanced relative to noise and the AUCs were improved. But if ${\beta }_{\mathrm{EP}}$ further increased to 70 or 90, the regularization became so strong that the true MCs were smoothed, resulting in lower AUCs. For a given ${\beta }_{\mathrm{EP}},$ decreasing ${\beta }_{\mathrm{RED}}$ meant a weaker regularization, resulting in higher image noise. On the other hand, reduced regularization placed greater emphasis on the data-fit term, thus enhancing the MC signals. This explains why we observed slight increase in AUC as ${\beta }_{\mathrm{RED}}$ decreased. We selected ${\beta }_{\mathrm{RED}}$ = 500 to balance the tradeoff between noise reduction and signal enhancement. In other parts of this study, we set the regularization parameter ${\beta }_{\mathrm{EP}}$ to 50 and ${\beta }_{\mathrm{RED}}$ to 500 if not specified.

### 3.3. MDR ablation study

We conducted an ablation study to illustrate the effects of the three terms in the MDR cost function (7). The three terms could be turned on or off separately to create 6 partial models. To turn off ${L}_{\mathrm{DBCN}}\left(x\right),$ we used the regular data-fit term ${L}_{\mathrm{regular}}\left(x\right)=\frac{1}{2}{\sum }_{i=1}^{{N}_{p}}{\unicode{x02016}{y}_{i}-{A}_{i}x\unicode{x02016}}_{2}^{2}$ instead of (4). ${R}_{\mathrm{EP}}\left(x\right)$ was turned off by setting ${\beta }_{\mathrm{EP}}$ to 0. ${R}_{\mathrm{RED}}\left(x\right)$ was turned off by setting ${\beta }_{\mathrm{RED}}$ to 0 and skipping all DNGAN denoising.

**Figure 6.**
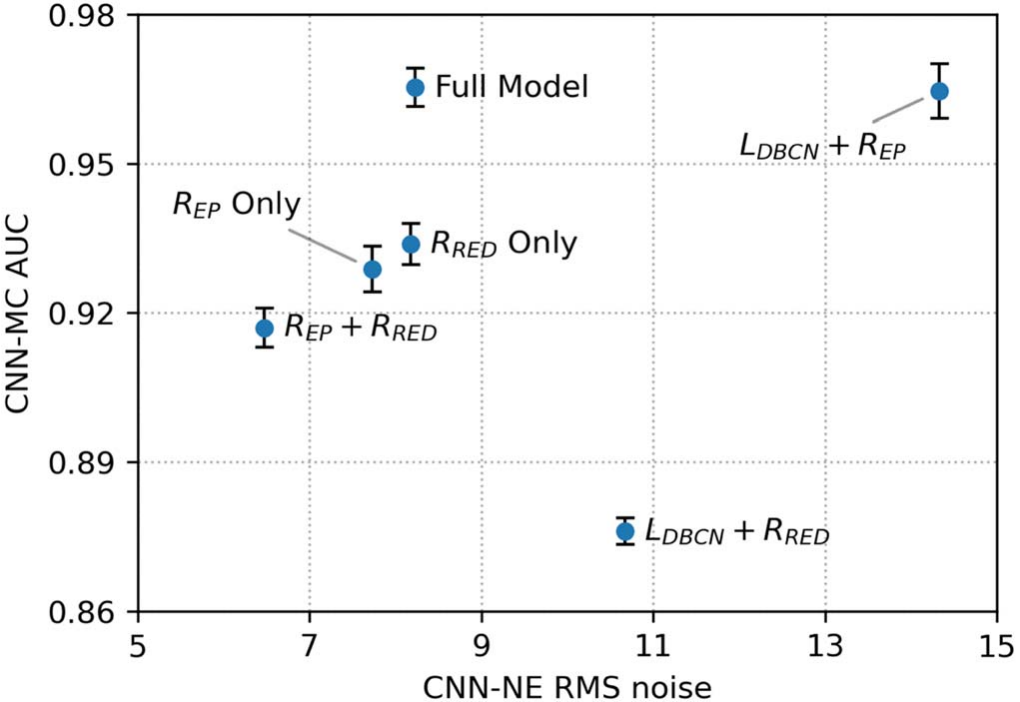
The task-based image quality assessment plot on the validation set showing the tradeoffs of the different terms in the MDR ablation study.

**Figure 7.**
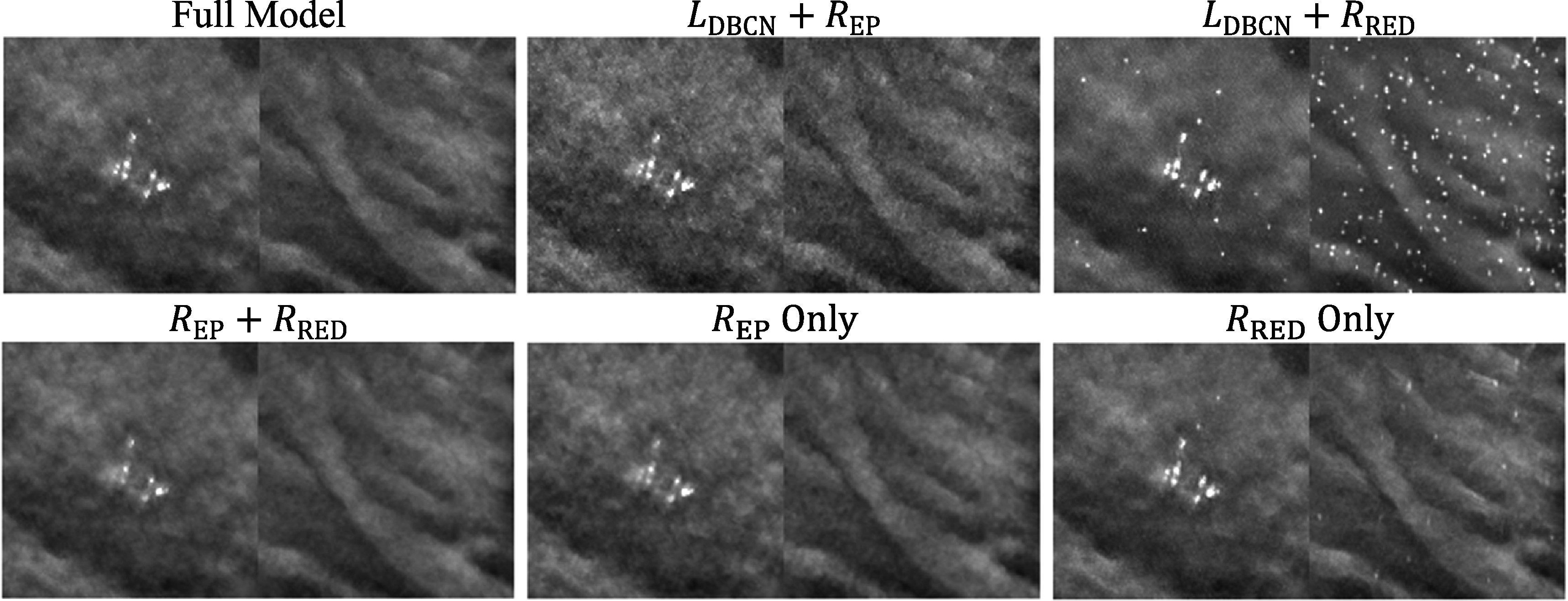
Example 3-slice MIP patches (12.8 mm × 12.8 mm) from the human subject validation set for the MDR ablation study. In each pair, a positive patch of MC cluster is shown on the left and a negative MC-free patch on the right.

Figure 6 shows the task-based image quality assessment plot for the VGG-Net backbone on the validation set for the different partial MDR models in comparison to the full MDR model. Figure 7 shows examples of MIP patches with and without MCs from the human subject validation set. From ‘${R}_{\mathrm{EP}}$ Only’, we found that the EP regularizer (${\beta }_{\mathrm{EP}}$ = 50) encouraged the smoothness of the images but sacrificed the sharpness of the MCs. The ${L}_{\mathrm{DBCN}}$ term contributed to signal enhancement, as can be seen from the AUC improvement between ‘${R}_{\mathrm{EP}}$ Only’ and ‘${L}_{\mathrm{DBCN}}+{R}_{\mathrm{EP}}$’, although it produced exceptionally noisy images if used alone. The ‘${L}_{\mathrm{DBCN}}$ Only’ condition (RMS noise: 101.1, AUC: 0.846 ± 0.004) is outside the plot range of figure 6 and is not shown in figure 7. The ‘${L}_{\mathrm{DBCN}}+{R}_{\mathrm{EP}}$’ condition had a high AUC but also a high RMS noise. From ‘${R}_{\mathrm{RED}}$ Only’, we observed that the RED regularizer (${\beta }_{\mathrm{RED}}$ = 500) powered by DNGAN was good at noise reduction and signal preservation, but sometimes it falsely enhanced the background tissue structures. Those MC-like enhancements were detected as false positives by CNN-MC and led to an overall low AUC. The false enhancement was even more severe for ‘${L}_{\mathrm{DBCN}}+{R}_{\mathrm{RED}}$’ which had the lowest AUC in figure 6. With ‘${R}_{\mathrm{EP}}+{R}_{\mathrm{RED}}$’, the false positives in the ‘${R}_{\mathrm{RED}}$ Only’ condition were suppressed and the RMS noise was the lowest, but it overly smoothed the MCs. Finally, the full MDR model took advantage of all three terms and achieved reasonably low RMS noise and the highest AUC.

### 3.4. Comparisons on validation set and independent test set

**Figure 8.**
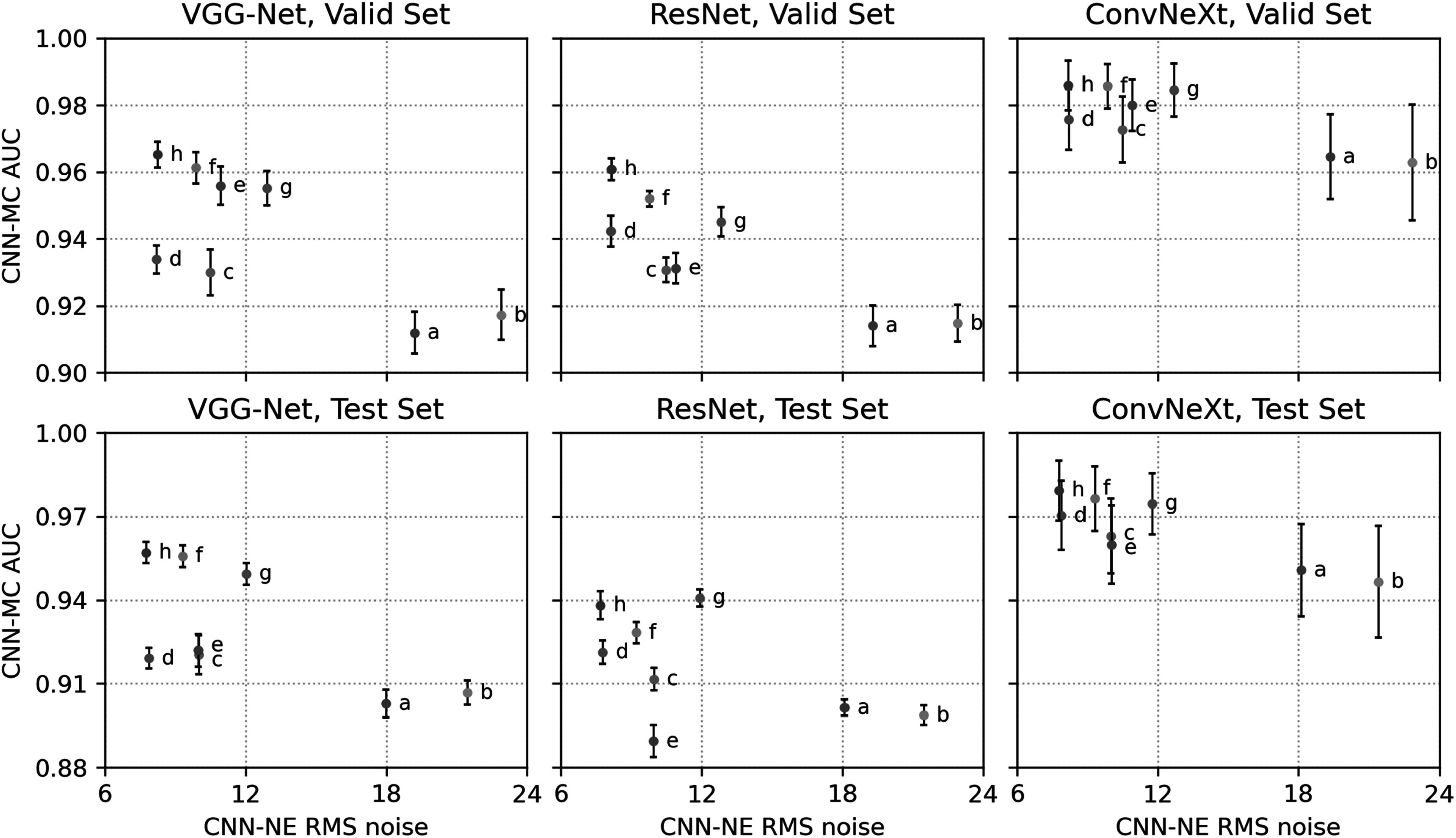
The task-based image quality assessment plot on the human subject validation set (first row) and test set (second row) using the VGG-Net (left column), ResNet (middle column) and ConvNeXt (right column) for comparing the reconstruction and denoising approaches. The labels (a) to (h) are defined in section 3.4 and table 3.

We compared eight image conditions in this section: (a) SART iteration 2; (b) SART iteration 3; (c) SART iteration 3 with post-reconstruction DNGAN denoising; (d) RED without DBCN modeling, equivalent to ‘${R}_{\mathrm{RED}}$ Only’ in section 3.3; (e) DBCN ($\beta $ = 70); (f) DBCN ($\beta $ = 50) with post-reconstruction DNGAN denoising; (g) MSBF; (h) MDR. Figure 8 shows the task-based image quality assessment plot for the validation set and the independent test set using the VGG-Net, ResNet and ConvNeXt backbones. The VGG-Net and ResNet had smaller error bars for AUCs and thus better reproducibility than ConvNeXt. The AUC and RMS noise rankings given by the three networks had some variations but were mostly consistent within the error bars. Table 3 summarizes the RMS noise and AUC ranking results in figure 8. The final rankings were obtained by summing the individual rankings. The rankings on the validation set largely agreed with those on the independent test set with minor variations between adjacent ranks except for DBCN, indicating reasonable generalizability of the proposed DCNN image quality assessment approach to the unseen test set.

**Table 3.** Summary of rankings for different reconstruction and denoising approaches using DCNN task-based image quality assessment in figure 8.

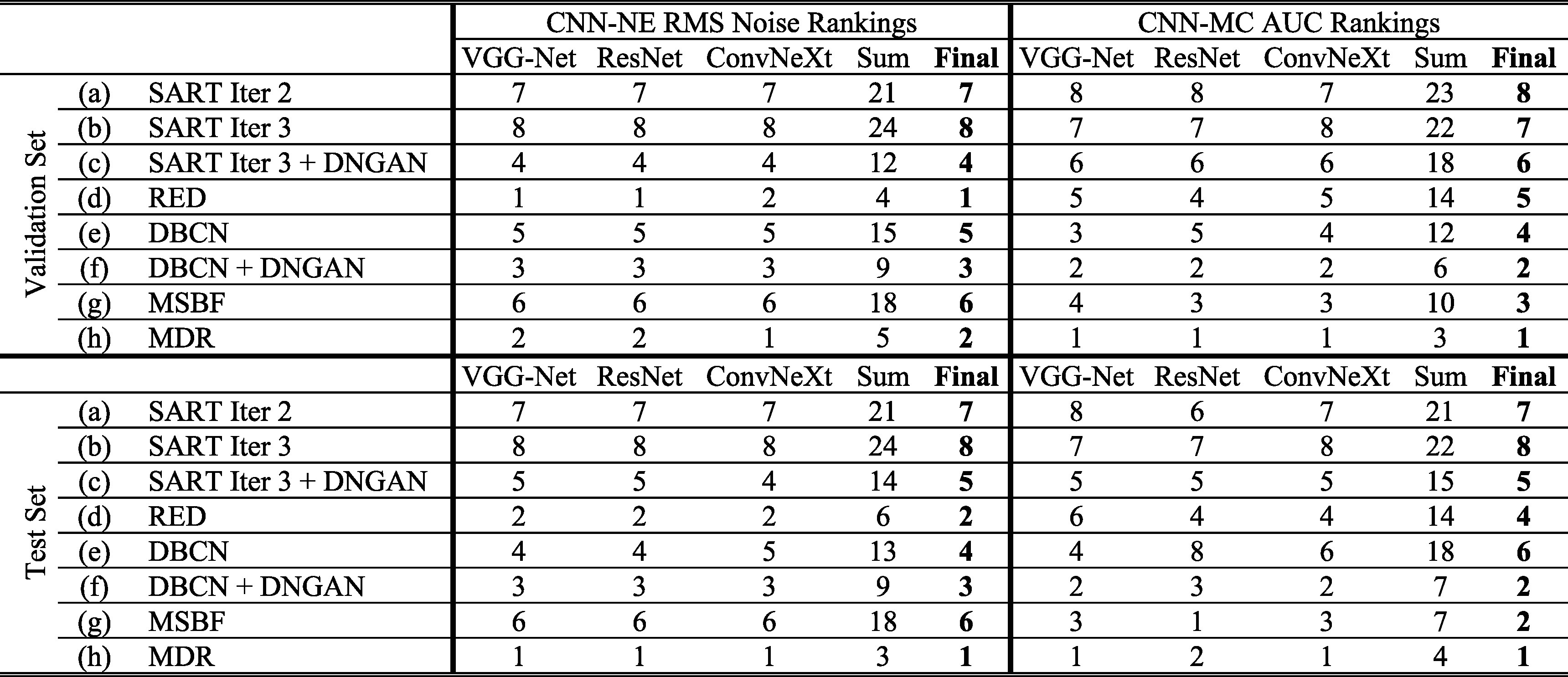

**Figure 9.**
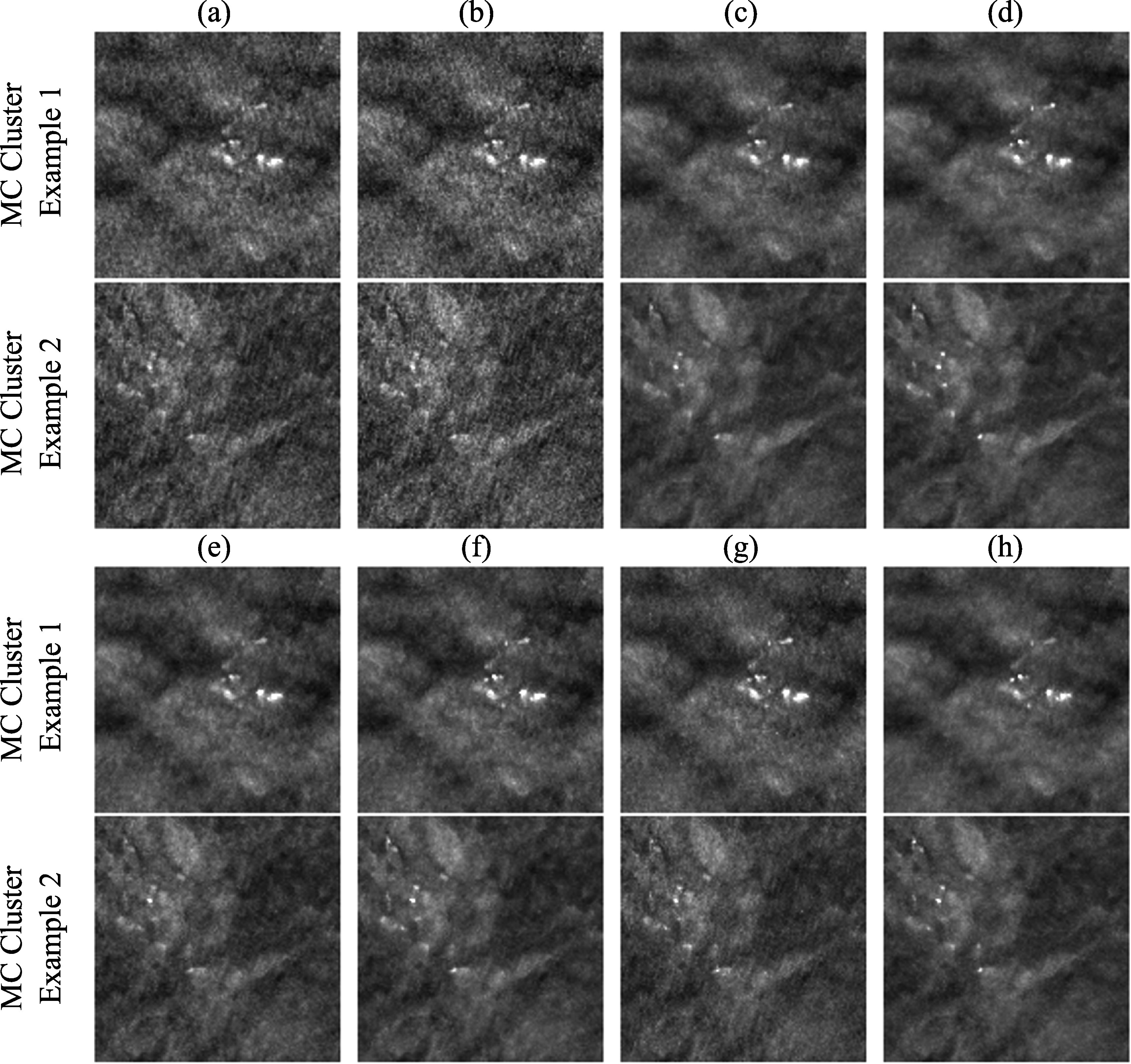
Example 3-slice MIP patches (12.8 mm × 12.8 mm) of two MC clusters (example 1: ductal carcinoma *in situ*; example 2: invasive ductal carcinoma) from the human subject test set. The labels (a) to (h) are defined in section 3.4 and table 3.

**Figure 10.**
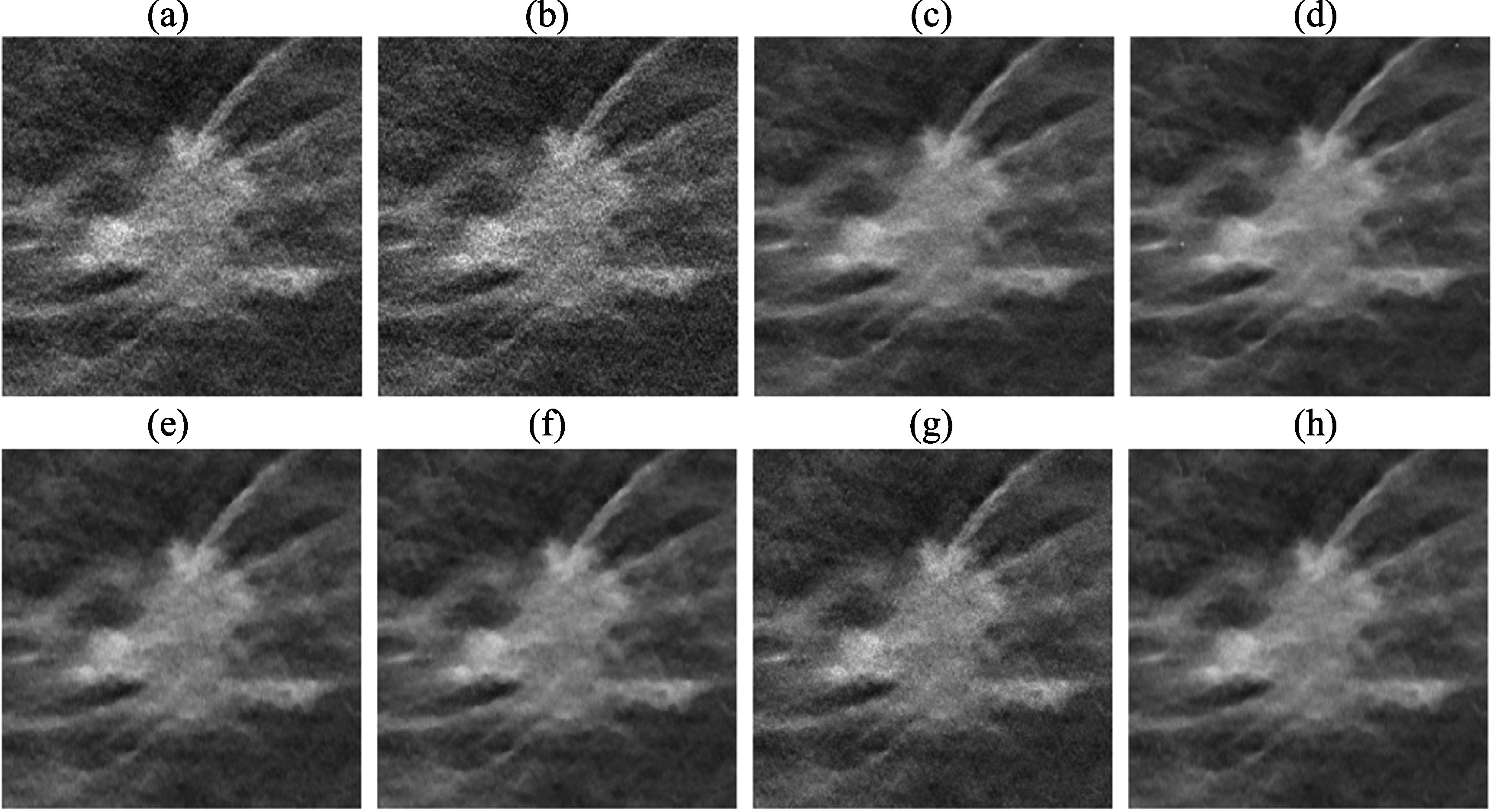
Example images (20 mm × 20 mm) of a spiculated mass (invasive ductal carcinoma) of a human subject DBT. The labels (a) to (h) are defined in section 3.4 and table 3.

Figure 9 shows the example MIP patches of MC clusters and figure 10 shows example images of a spiculated mass from the human subject test set. The visual judgment of the noise levels matched the quantitative RMS noise estimation. The SART images (conditions (a) and (b)) were noisy with poor MC conspicuity as it is an unregularized reconstruction method (Jiang and Wang 2003). Increasing the number of SART iterations enhanced the signal, but it also amplified noise, resulting in comparable signal-to-noise ratios and thus AUCs, as illustrated in figure 8. This observation reaffirmed our understanding of SART that there was no benefit of continuing SART iterations without regularization. Furthermore, it highlighted the advantage of our task-based metric using CNN-MC, which focused on the detectability of the MC clusters and provided a more informative assessment. DBCN (condition (e)) had relatively large variations of AUC rankings among the three CNN-MCs and two data sets, possibly because its patchy and noisy background confused the image features and made the training set less representative of the data distribution. Post-reconstruction denoising with DNGAN was flexible for application to different reconstruction techniques, reducing image noise and improving the AUC as shown in the examples for both SART (condition (c) in comparison to (b)) and DBCN (condition (f) in comparison to (e)). Note that, with DNGAN denoising, SART and DBCN could use parameters of stronger MC enhancement (3 iterations instead of 2 in SART, and $\beta $ = 50 instead of 70 in DBCN). Although RED (condition (d)) reached a very low noise level and its visual signal quality was comparable to that of MDR, its AUC was low due to false enhancement as discussed in section 3.3. The conspicuous bright spots resembling MCs in the examples shown in figure 7 (lower right) and figure 10(d) reiterated this downside of RED. The AUC ranking of MSBF (condition (g)) was high, but its noise also remained high. The proposed MDR (condition (h)) achieved one of the lowest RMS noise and the highest AUC rankings than other image conditions. The high visibility of the subtle MCs and smooth background of the MDR images can also be clearly seen in figure 9. Moreover, it preserved the fine texture details and had a satisfactory visual quality of mass margins without creating artifact in figure 10.

### 3.5. Qualitative comparison between MDR and commercial reconstruction

**Figure 11.**
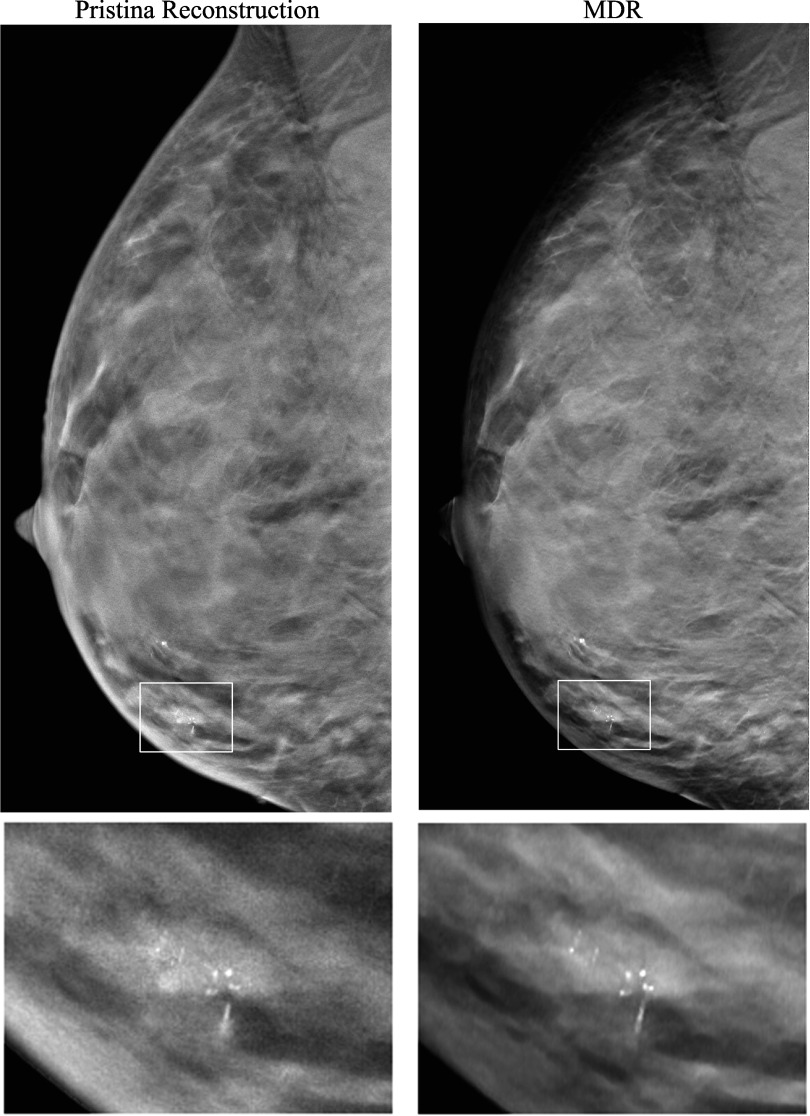
Qualitative comparison between the commercial GE Pristina reconstruction and the proposed MDR. Top row: full DBT slices (84.4 mm × 170 mm). Bottom row: zoomed-in views of the marked regions (20 mm × 15 mm) containing an MC cluster (ductal carcinoma *in situ*).

The GEN2 DBT system used for acquiring the human subject DBTs was developed as a prototype for research purposes, so it lacked a built-in reconstruction method. To compare the proposed MDR with gold standard reconstruction, we reconstructed a human subject DBT case acquired with the commercial GE Pristina DBT system. Figure 11 shows a qualitative comparison of the full DBT slices and the zoomed-in views of an MC cluster obtained with the Pristina reconstruction and our MDR approach. The full slice image of MDR had reduced noise levels compared to the Pristina reconstruction. The zoomed images facilitated the visualization of the nuances of the MC signals, surrounding tissue structures, and noise. The MDR not only achieved a substantial reduction in noise, but also preserved details and enhanced the subtle MC specks, demonstrating superior image quality than the commercial reconstruction.

The darkening of breast periphery as seen in the MDR reconstructed image in figure 11 resulted from the physics of x-ray imaging. The reduction in tissue thickness and thus increasing penetration of x-ray exposure to the detector caused varying image intensities near the breast boundaries. Commercial DBT images are generally processed with peripheral equalization software to facilitate radiologists’ reading. We did not attempt to address this issue because we did not have access to any breast peripheral equalization software and its development was outside the scope of this study. One can always view the peripheral regions by adjusting the display contrast and brightness properly. As demonstrated in figure 11, the proposed MDR method enhanced the MC signals regardless of their location. Despite being near the breast periphery, the enlarged ROI still shows the improvement of image quality by the MDR.

## 4. Discussion

DBCN and DNGAN represent two distinct approaches for enhancing the subtle signals in DBT. The DBCN method approximates the deblurring and noise decorrelation by the inverse of the detector point spread filter and the prewhitening filter. Both operations boost the high frequency signals, which inevitably amplify high frequency noise at the same time. DBCN therefore is incorporated with an EP regularizer to control the noise. DNGAN emphasizes the signals by smoothing the surrounding backgrounds like a regularizer. It also increases the contrast of the signals to some extent, but sometimes falsely enhances the high frequency structures and creates MC-like artifacts. We explored combining the two methods to take advantage of both. We implemented the newly proposed MDR method in an efficient way so that it could reconstruct the full-sized 3D DBT images at full resolution. Quantitative results using our newly developed DCNN image quality measures on the independent test set of human subject DBTs revealed that MDR achieved the highest AUC rankings for MC detectability and low noise among the reconstruction and denoising approaches studied. This study shows the promise of combining the physics-based MBIR and the learning-based DCNNs for reconstruction.

The CNN-MC model observer can characterize the detectability of the MC clusters in human subject DBTs. Because of the complexity of the SKS MC clusters, we trained the CNN-MC to learn the signal representations through back-propagation, unlike CHO and *d’* where the target signal is trained to be fitted with channelized functions or mathematically defined. Moreover, the CNN-MC can include the assessment of image background with and without MC-like false enhancement (shown in section 3.3) that the traditional image quality measures such as *d’* cannot achieve because they focus on individual signals. Assessing potential false information generated by an image processing method is crucial for evaluating its feasibility in clinical applications.

The role of CNN-NE can be understood from two aspects. First, CNN-NE played a pivotal role in our training and processing pipeline because it served as the pre-trained model of CNN-MC, which worked well only when transfer-learned from CNN-NE (figure 4). The advantages of transfer learning were elaborated in section 2.2.3. Second, despite the possibility of analytically calculating the RMS noise of an image patch, our work showcased the ability of deep CNN to approximate this calculation. There is also a growing interest in applying CNNs for noise quantification in medical imaging (Huber *et al* 2023). The development of CNN-NE provides users the flexibility of choosing between analytical and CNN calculations for estimating the RMS noise. It is noteworthy that CNN-NE has superior computational efficiency when run on a GPU, compared to the time-consuming grid-based calculation method. We proposed to use CNN-NE and CNN-MC together to guide the selection of the reconstruction parameters and compare different reconstruction techniques and denoising approaches. The task-based image quality assessment plot of CNN-MC AUC versus CNN-NE RMS noise clearly illustrates the tradeoff between the detectability and noise of the different image conditions.

The RMS noise calculation introduced in section 2.2.2 involved a quadratic fitting step to remove the background trend. Given that we used VICTRE simulation data for CNN-NE training and simulations can produce (nearly) noise-free images, it is possible to remove the background structures using such noise-free images. To verify that the RMS noise in images could be effectively estimated by the quadratic fitting background removal method, we simulated the nearly noise-free images using an exposure factor of 10, and then calculated the RMS values for the CNN-NE training image patches with the exposure factor of 1 using the two approaches. The result showed that the two sets of RMS values were close and exhibited a strong linear correlation (correlation coefficient = 0.983, *p* < 0.0001), indicating the effectiveness of quadratic fitting for background removal. While using noise-free images may be the ideal approach for accurate RMS calculations, its real-world impact is limited due to the linearity and small differences. More importantly, having the noise-free images is a special case and they are typically unavailable for actual scanned images. Therefore, we employed the fitting method in our work due to its practicality in general scenarios in case others would want to adopt the method for their applications.

A suboptimal CNN-MC model observer is preferred for evaluating the effectiveness of image processing techniques since image processing is not expected to improve the performance of an ideal observer. Zhang *et al* (2021) showed that a DCNN could be trained to approximate an ideal observer or a suboptimal observer, depending on its learning capacity and the available training sample size, for the task of detecting MC clusters in a simulation study. This is consistent with the observations in our study. We found that DCNNs of very deep structures reached an AUC of 1 regardless of the image conditions. We had to constrain the learning capacity by limiting the network sizes such that they could discriminate between the image conditions. In addition, it is known that human observers are suboptimal, as also demonstrated by Chan *et al* (2023). Since there are no specific guides for selecting the DCNN structure for a given task, we investigated three CNN-MC backbones. The pooled performance of the multiple models may partly alleviate the learning variability, in analogy to the use of multiple observers to account for the interobserver variability in human reader studies. The CNN-NE trained as a regression model was much more stable, as observed from the small variances among the different initializations and different CNN backbones.

We did not analyze the convergence property of MDR in this study. Due to the limited-angle nature of the DBT scan and the fact that radiologists are accustomed to the tissue texture appearance of the 2D projection mammograms, we usually do early stopping on the DBT reconstruction so that the soft tissue structured backgrounds are not overly enhanced. The truncation artifact correction (Lu *et al* 2013) during reconstruction essentially changes the system matrices and also complicates the convergence analysis. Furthermore, the RED assumptions are not satisfied by many popular denoisers including DNGAN (Reehorst and Schniter 2019). Without the explicit expression and minimization of a cost function, RED serves only as a motivation and one can at best hope to obtain the fixed-point convergence or an equilibrium of the reconstruction (Buzzard *et al* 2018, Ryu *et al* 2019). For these reasons, we focused on image quality instead of seeking the convergence of the iterates.

There are limitations in the current study. First, the DNGAN denoisers were trained on the SART images but were directly applied to the intermediate images of MDR. This introduced a noise mismatch between the denoiser and the images to be denoised. The fact that the DNGAN works well in MDR indicates the flexibility of the application, but it also leaves room for improvement by fine-tuning the DNGAN denoisers at every MDR iteration, which will tradeoff computational efficiency, however. Second, the number of MC clusters in our data set is limited. A larger CNN-MC training set may further improve the generalizability of the CNN-MC model observer and reduce the AUC variations among the different DCNN models or between training and deployment to unseen cases. Third, although MDR achieved the highest MC detectability ranking and low noise, post DBCN reconstruction with DNGAN denoising was also among the top three rankings. Post-reconstruction denoising is the only practical approach if the raw PVs are not available for user-designed reconstruction. It will be of interest to evaluate the effectiveness of DNGAN denoising for the DBT images reconstructed by the various commercial DBT systems when large human subject data sets with subtle MCs from these systems become available. Fourth, the proposed DCNN image evaluation approach only focused on image noise and MC detectability. The evaluations of mass and tissue textures still relied on visual judgment. It will be useful if DCNN models can be developed to assess the quality of these important image features as well in the future. Finally, we do not address the time efficiency of MBIR as it falls outside the primary scope of this paper. We encourage future research to tackle this issue, which may complement and enhance the utility of the proposed method.

## 5. Conclusion

We proposed MDR by combining DBCN modeling and DNGAN denoising using the RED framework for DBT reconstruction. To facilitate the task-based image quality assessment, we also proposed two DCNN tools. The CNN-NE was trained to estimate the RMS noise of the DBT images. The CNN-MC was trained to be a model observer to evaluate the detectability of clustered MCs. The efficacies of DNGAN, CNN-NE and CNN-MC were demonstrated using physical phantom DBTs and human subject DBTs. We investigated the impacts of the MDR regularization parameters and the cost function terms. MDR reduced image noise and improved MC detectability on an independent test set of human subject DBTs. The proposed CNN-NE and CNN-MC evaluation method can serve as a surrogate for human observers to provide task-specific metrics and rank the imaging systems in a cost-effective way. The proposed reconstruction method may potentially lead to lower dose and higher sensitivity and specificity for MC detection in breast cancer screening and diagnosis.

## Data Availability

The data cannot be made publicly available upon publication because the cost of preparing, depositing and hosting the data would be prohibitive within the terms of this research project. The data that support the findings of this study are available upon reasonable request from the authors.
